# Antimicrobial peptide for bacterial infection imaging: first case reported in Brazil

**DOI:** 10.31744/einstein_journal/2023RC0621

**Published:** 2023-11-23

**Authors:** Solange Amorim Nogueira, Marycel Rosa Felisa Figols de Barboza, Rosemeire Pereira Bezerra, Jorge Mejia Cabeza, Adriana Macedo Dell’Aquila, Durval do Carmo Barros Santos, Lilian Yuri Itaya Yamaga, Akemi Osawa

**Affiliations:** 1 Hospital Israelita Albert Einstein Department of Radiology and Diagnostic Imaging São Paulo SP Brazil Department of Radiology and Diagnostic Imaging, Hospital Israelita Albert Einstein, São Paulo, SP, Brazil.; 2 Hospital do Servidor Público Estadual de São Paulo Department of Medicine and Infectious Diseases São Paulo SP Brazil Department of Medicine and Infectious Diseases, Hospital do Servidor Público Estadual de São Paulo, São Paulo, SP, Brazil.

**Keywords:** Osteomyelitis, *Staphylococcus aureus*, *Staphylococcal infections*, Radiopharmaceuticals, Antimicrobial peptides, Positron emission tomography computed tomography, Bacterial infections

## Abstract

Molecular imaging markers can be used to differentiate between infection and aseptic inflammation, determine the severity of infection, and monitor treatment responses. One of these markers is ubiquicidin(29-41) (UBI), a cationic peptide fragment that binds to the bacterial membrane wall and is labeled with gallium-68 (^68^Ga), a positron emitter radioisotope. The use of UBI in positron emission tomography (PET)/computed tomography (CT) for improved detection of lesions has been receiving considerable attention recently. Herein, we report the first case of ^68^Ga-UBI PET/CT performed in Brazil. The patient was a 39-year-old woman referred for a scan to confirm a clinical suspicion of chronic osteomyelitis of her fractured left tibia. PET images revealed radiotracer uptake near the posterior contour of the tibial fracture focus and the fixation plate, in the soft tissue around the distal half of the tibia, and in the non-consolidated fracture of the left distal fibula. Surgery for local cleaning was performed, and culture of a specimen collected from the surgical site confirmed the presence of *Staphylococcus aureus*. In the present case, ^68^Ga-UBI PET/CT, a non-invasive imaging modality, identified the infection foci *in vivo*, indicating its potential for clinical use.

## INTRODUCTION

Distinguishing between infection and aseptic inflammation is clinically challenging. Molecular imaging markers can be used accurately assess the extent of disease, identify infection sites, and track treatment responses.^(1,2)^

In nuclear medicine, ^67^Ga-Citrate and glucose analog (^18^F-FDG) imaging can be used to locate infection sites. However, gallium behaves similarly to iron and binds to transferrin, a protein involved in the inflammatory response.^(2)^ Moreover, PET/CT with ^18^F-FDG cannot differentiate infection from tumor cells and aseptic processes such as inflammation. This is because cells such as neutrophils, macrophages, and activated leukocytes show high expression levels of glucose transporters. Circulating cytokines increase the affinity of these transporters, leading to increased ^18^F-FDG uptake.^(2-5)^

Ubiquicidin(29-41) (UBI) is a cationic peptide fragment that binds to bacterial cell membranes through a specific binding mechanism that does not rely on leukocyte function. When labeled with a radioisotope, such as gallium 68 (^68^Ga), UBI can be used as a radiotracer.^(4,5)^

^68^Ga-UBI is hydrophilic, which means that it is absorbed heavily by the kidneys, urinary tract, and bladder, and easily excreted. The biodistribution of ^68^Ga-UBI is characterized by minimal uptake in organs and tissues. Therefore, any increase in tracer uptake outside the usual biodistribution areas is considered a positive indicator of an infectious process.^(6)^

*Hospital Israelita Albert Einstein* São Paulo, Brazil, recently implemented the protocol for the synthesis and quality control of ^68^Ga-UBI (using dodecane tetraacetic acid [DOTA] as a chelate) in the hospital radiopharmacy for clinical use in the nuclear medicine department. Herein, we report the first case of ^68^Ga-UBI PET/CT performed in Brazil.

## CASE REPORT

A 39-year-old female patient fractured her left tibia and fibula and underwent osteosynthesis for repair of the fracture. However, osteomyelitis occurred postoperatively, and the patient was placed on a ten-day course of cephalexin. Two months after the surgery, the patient experienced a fall and underwent an additional surgery for insertion of support plates and screws in the tibia and fibula. Signs of infection were observed after the surgery; thus, a three-month course of antibiotic therapy that included ciprofloxacin and bactrim was initiated. Despite completing the treatment, the patient continued to experience discomfort and reported observing a little pus at the surgical site. Further testing revealed the presence of *Enterobacter cloacae* in the patient's blood culture, prompting a referral for a ^68^Ga-UBI PET/CT to confirm a potential infectious process and suspected osteomyelitis in the left tibia.

A ^68^Ge/^68^Ga generator (IGG-100) and an automated module (Modular-Lab Pharm Tracer; Eckert and Ziegler) were utilized for the synthesis of ^68^Ga-UBI. The DOTA-UBI was obtained from ABX.

Whole-body PET/CT was performed 60 minutes after intravenous administration of ^68^Ga-UBI (260MBq) using a Biograph mCT 40 PET/CT scanner (*Siemens Healthineers*). The parameters for this protocol included an imaging time of 4 minutes per bed position, ultra-HD-PET reconstruction, a matrix of 200x200, two iterations, and 21 subsets. No contrast agent was used.

**Figure 1:**
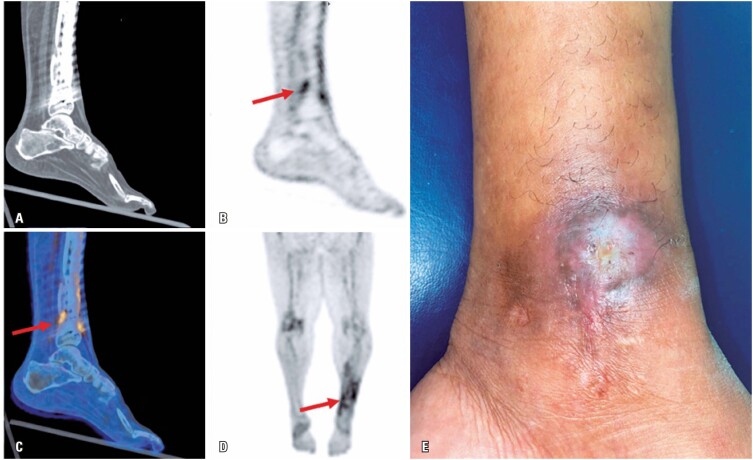
Computed tomography (A), PET (B) and fusion (C) images in sagittal plane; and image of maximum intensity projection of the tibia region (D); the red arrows indicate the ^68^Ga-UBI uptake in infection foci, and image of injury in the surgical scar on the tibia's distal region (E)

Figure 1 shows PET/CT images illustrating ^68^Ga-UBI uptake in the periosteal region of the left tibia, in both the diaphysis and metaphysis. The uptake was primarily observed near the posterior contour of the tibial fracture focus and the fixation plate (standardized uptake value [SUV], 2.8), in the soft tissue around the distal half of the tibia, and in the non-consolidated fracture of the left distal fibula, which was fixed with a lateral plate and screw. The SUV of the uptake in the non-consolidated fracture of the left distal fibula was 2.5, and the uptake was predominantly near the screw inserted immediately above the fracture. After the examination, the patient underwent another surgery performed to clean the osteomyelitis foci. Culture of a specimen collected from the surgical site confirmed the presence of *Staphylococcus aureus*.

This case report is part of a study approved by the Research Ethics Committee of *Hospital Israelita Albert Einstein* under CAAE: 47052521.9.0000.0071; # 5.824.299.

## DISCUSSION

The identification of bacteria at lesion sites using noninvasive procedures is challenging. Some radiopharmaceuticals have been specifically developed to distinguish between infectious and aseptic processes. UBI labeled with a radioisotope is among the precursors studied for this purpose.^(1)^

UBI is a cationic peptide fragment that attaches to negatively charged bacterial membranes. This fragment, formerly labeled with technetium-99m, aids the identification of infections on noninvasive scintigraphy images.^(7,8)^ There is growing interest in the use of UBI in PET/CT, with the aim of taking advantage of the superior sensitivity and spatial resolution of the imaging modality to improve the detection of lesions.^(2)^

In 2018, Ebenhan et al. described the hydrophilic behavior of UBI linked to the chelator NOTA and labeled with ^68^Ga. This is the reason underlying the increased uptake of ^68^Ga-UBI in the kidneys, urinary tract, and bladder due to excretion. The biodistribution of ^68^Ga-UBI is characterized by minimal uptake in tissues and organs,^(6)^ which was observed in the use of UBI with DOTA as a chelator in the present case.

A few casuistic studies have demonstrated that ^68^Ga-UBI PET/CT differentiates between infectious and inflammatory processes. A systematic review of articles published in 2023 provided substantial evidence that ^68^Ga-UBI is a selective and specific radiopharmaceutical that could identify bacterial infections.^(8)^ This finding indicates that ^68^Ga-UBI could facilitate the differentiation of infection from aseptic inflammation in clinical settings.^(8-10)^

## CONCLUSION

This report describes the first use of ^68^Ga-UBI PET/CT in Brazil to identify bacterial infection sites. Further studies are required to determine the accuracy and sensitivity of this radiotracer in distinguishing between infectious sites and aseptic processes.
